# Climate Change Across Seasons Experiment (CCASE): A new method for simulating future climate in seasonally snow-covered ecosystems

**DOI:** 10.1371/journal.pone.0171928

**Published:** 2017-02-16

**Authors:** Pamela H. Templer, Andrew B. Reinmann, Rebecca Sanders-DeMott, Patrick O. Sorensen, Stephanie M. Juice, Francis Bowles, Laura E. Sofen, Jamie L. Harrison, Ian Halm, Lindsey Rustad, Mary E. Martin, Nicholas Grant

**Affiliations:** 1 Boston University, Department of Biology, Boston, Massachusetts, United States of America; 2 Research Designs, Lyme, New Hampshire, United States of America; 3 US Forest Service, Hubbard Brook Experimental Forest, Woodstock, New Hampshire, United States of America; 4 University New Hampshire, Institute for the Study of Earth, Oceans, and Space, Durham, New Hampshire, United States of America; WSL Institute for Snow and Avalanche Research SLF, SWITZERLAND

## Abstract

Climate models project an increase in mean annual air temperatures and a reduction in the depth and duration of winter snowpack for many mid and high latitude and high elevation seasonally snow-covered ecosystems over the next century. The combined effects of these changes in climate will lead to warmer soils in the growing season and increased frequency of soil freeze-thaw cycles (FTCs) in winter due to the loss of a continuous, insulating snowpack. Previous experiments have warmed soils or removed snow via shoveling or with shelters to mimic projected declines in the winter snowpack. To our knowledge, no experiment has examined the interactive effects of declining snowpack and increased frequency of soil FTCs, combined with soil warming in the snow-free season on terrestrial ecosystems. In addition, none have mimicked directly the projected increase in soil FTC frequency in tall statured forests that is expected as a result of a loss of insulating snow in winter. We established the Climate Change Across Seasons Experiment (CCASE) at Hubbard Brook Experimental Forest in the White Mountains of New Hampshire in 2012 to assess the combined effects of these changes in climate on a variety of pedoclimate conditions, biogeochemical processes, and ecology of northern hardwood forests. This paper demonstrates the feasibility of creating soil FTC events in a tall statured ecosystem in winter to simulate the projected increase in soil FTC frequency over the next century and combines this projected change in winter climate with ecosystem warming throughout the snow-free season. Together, this experiment provides a new and more comprehensive approach for climate change experiments that can be adopted in other seasonally snow-covered ecosystems to simulate expected changes resulting from global air temperature rise.

## Introduction

More than 45% of land area in the northern hemisphere is currently affected by seasonal snow cover and 57% is affected by soil freezing [1]. Climate models project an increase in air temperatures and reductions in snow depth and duration over the next century that will affect soil thermal regimes throughout many of these ecosystems [2]. Snow cover insulates soils from below-freezing air temperatures [3, 4, 5]; therefore, a reduced snowpack is projected to lead to colder soils in winter with increased frequency of freeze-thaw cycles in many ecosystems (FTCs; [5, 6, 7]). Many multi-factor experiments have examined the combined effects of various environmental changes simultaneously, including elevated CO_2_, nitrogen availability, and temperature [8]. Other studies have evaluated in isolation the effects of warmer temperatures [9] or the effects of changes in winter climate through snow manipulations [10], but the interactive effects of reduced winter snow cover and increased frequency of soil FTC, combined with warming in the snow-free season are not well understood.

There are a variety of ways researchers warm ecosystems experimentally, including passively through open top chambers (e.g., [11]), glasshouses (e.g., [12]), and infrared reflecting curtains [13] or actively through buried heating cables (e.g., [14]), infrared lamps (e.g., [15]) or active heating chambers (e.g., [16]) aboveground. In short-statured ecosystems such as grasslands and tundra, infrared heaters, open top chambers and greenhouses are often used to warm soil and air temperatures. Although aboveground warming most realistically mimics projected increases in air temperature [17], obtaining consistent aboveground warming in tall statured forests such as forest ecosystems is challenging both logistically and financially, thus buried heating cables are commonly used for warming experiments in forest ecosystems [9, 18].

An increasing number of studies recognize the importance of evaluating changes in winter climate [19]. Previous studies have examined the effects of a smaller snowpack on the functioning of terrestrial ecosystems primarily through snowmelt induced by infrared lamps in short-statured ecosystems such as tundra and grasslands [20, 21] or snow-removal via shoveling [22, 23] or addition of water to mimic rain on snow events [24] in tall statured ecosystems such as forests. Many snow removal experiments in the field induce persistent soil freezing (e.g., [22, 25]), while fewer induce FTCs in soil (e.g., 20, 26]). The distinction between the two forms of soil freezing is important since many ecosystems that have historically experienced snow are likely to experience an increase in FTCs as air temperatures oscillate around the freezing point, rather than a longer duration of soil freezing, over the next 100 years [5, 6]. Most experiments that purposefully induce soil FTCs are limited to laboratory experiments (e.g., [27]) and/or create severe freeze/thaw conditions that may be more intense than ecosystems experience in reality [5, 28, 29]. While some studies have mimicked soil FTCs in the field under realistic conditions, these have been limited to ecosystems dominated by short-statured grasses and/or shrub species (e.g., [20, 26]). To our knowledge no study has utilized a combination of snow-removal and buried heating cables to induce soil FTCs in a mature forest ecosystem and none have combined this experimental treatment with warming during the snow-free season to examine the interactive effects of climate change across seasons in a terrestrial ecosystem. The “across season” effects could be antagonistic, additive, or synergistic, which would not be apparent from examining one season alone.

We designed and implemented the Climate Change Across Seasons Experiment (CCASE) at Hubbard Brook Experimental Forest in the White Mountains of New Hampshire, USA to (1) determine the feasibility of inducing soil FTCs in winter in a tall statured forest ecosystem and (2) to examine the combined effects of increased frequency of soil FTCs in winter with warming in the snow-free season on a variety of forest biota and ecosystem processes. We mimicked the projected increase in temperatures and soil FTC frequency for this region over the next century [6] to make results of this experiment realistic and relevant for understanding and predicting future trends in northern forest ecosystems. This paper does not address specific hypotheses, but rather lays the framework and demonstrates the feasibility of creating experiments in seasonally snow-covered forest ecosystems to both induce a greater frequency of soil FTCs and examine its interaction with warmer temperatures in the snow-free season. Our experiment fills a critical gap in research and will improve our understanding of the response of terrestrial ecosystems to global temperature rise.

## Materials and methods

### Study site

The CCASE experiment is located at Hubbard Brook Experiment Forest (HBEF) in the White Mountain National Forest in Central New Hampshire, USA (43°56’N, 71°45’W). Permission to conduct this research at the Hubbard Brook Experimental Forest was provided by employees of the US Forest Service. Hubbard Brook is a National Science Foundation Long-Term Ecological Research site. Canopy vegetation is primarily dominated by northern hardwoods. The prevalent hardwood tree species include red maple (*Acer rubrum*), sugar maple (*Acer saccharum*), American beech (*Fagus grandifolia*), and yellow birch (*Betula alleghaniensis*); conifer species include red spruce (*Picea rubens*), eastern hemlock (*Tsuga canadensis*), white pine (*Pinus strobus*) and balsam fir (*Abies balsamea*). Soils consist of Typic Haplorthods (Scott Bailey, *personal communication*), which developed in glaciofluvial sand and gravel; bedrock is approximately 14 m deep [30]. The climate is cool, humid, and continental. Mean annual precipitation is 1400 mm with approximately one-third falling as snow [31]. The winter snowpack typically lasts from mid-December to mid-April and winter air temperatures average -4.7°C [6]. Soil frost is present approximately two out of every three years, with an average annual maximum depth of 6 cm from the surface of the forest floor [6]. During the last half-century, winter air temperatures at HBEF have risen by 2.5°C, the maximum depth of winter snowpack has declined by 26 cm, and the duration of winter snow cover has declined by four days per decade [31, 32, 33].

### Experimental design

**Fig 1:**
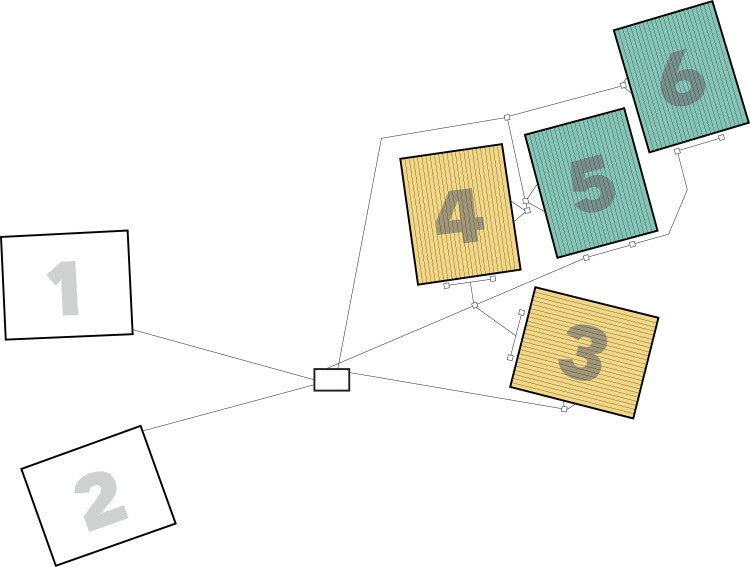
Diagram of reference and heated plots, including wiring and conduit systems. Each plot is 11 X 13.5 m^2^. Plots 1 and 2 are *reference*, 3 and 4 are *warmed*, and 5 and 6 are *warmed +FTC*. EPDM synthetic rubber roofing membrane material was installed to 30 cm depth between the two types of warmed plots (i.e., between plots 3–4 and 5–6) to inhibit roots from growing into different treatments. The parallel gray lines inside plots represent heating cable and the black lines outside plots represent heating cable conduit. The junction boxes, located along the conduit, are represented by white boxes. The equipment shed (center box) houses the control equipment for the experiment.

The CCASE experiment consists of six plots (each 11 X 13.5 m^2^), each centered on a minimum of three mature red maple trees (Fig 1). Two plots serve as reference (hereafter referred to as “*reference*”), two have soils warmed 5°C throughout the snow-free season (hereafter referred to as “*warmed*”), and two others have soils warmed 5°C throughout the snow-free season with soil FTCs induced in winter (hereafter referred to as “*warmed + FTC*”). All six plots are dominated by mature red maple trees, which make up 63.1 ± 6.9% basal area with an understory composed of mostly American beech (Table 1). The depth of the organic layer is on average 5.2 ± 0.3 cm and soils in this experiment are classified as sand and loamy sands (M. Vadeboncoeur, *personal communication*). We purposefully located the plots to have similar basal area of individual tree species and total basal area across all tree species among plots (Table 1). We did not randomly assign treatments across the six plots because of logistical constraints associated with electrical infrastructure. Future studies should take into account the trade-offs between replication of experimental treatments and size of individual plots given the high cost of both infrastructure and energy required to heat the plots. It would have been ideal in this experiment to have a greater number of plots per treatment. Because our goal was to evaluate both below- and aboveground responses to changes in temperature in this tall statured ecosystem, we chose to have relatively large plots that were replicated only twice.

**Table 1:** Total basal area of trees in each of the six CCASE plots. Values are totals per tree species within each plot measured in June 2012. Units are cm^2^. There were no statistically significant differences in total basal area of individual tree species comparing reference (plots 1 and 2) to treatment (plots 3–6) plots (*P* > 0.05 for all tree species; see below) or for total basal area across all tree species comparing reference to treatment plots (*P* = 0.91).

	Plot 1	2	3	4	5	6	* *
Tree Species	Reference	Reference	Warmed	Warmed	Warmed	Warmed	*P* value
					+ FTC	+ FTC	
*Acer rubrum*	3395	2043	2502	3973	2007	3277	0.79
*Fagus grandifolia*	1922	762	765	386	429	636	0.11
*Betula papyrifera*	0	0	332	0	0	0	0.54
*Betula alleghaniensis*	62	0	89	1734	0	0	0.54
*Acer saccharum*	0	0	0	201	69	580	0.34
*Tsuga canadensis*	0	0	43	0	0	0	0.54
*Fraxinus americana*	0	52	0	0	248	0	0.72
*Picea rubens*	0	0	0	0	0	32	0.54
*Populus grandidentata*	0	701	0	0	0	0	0.18

We buried a total of 4 km of resistance heating cables in the *warmed* and *warmed + FTC* plots in July and August 2012. Cable installation required approximately 340 person hours over three weeks to complete. Soil heating cables were buried 10 cm deep in lines that are 20 cm apart, making a total of 56 lines of cable across each plot (Fig 1). We used a flat-blade, square-edge shovel to make a 10 cm vertical cut in the soil for each cable, then used drywall spatulas to open the soil and gently insert the cable by hand. The cable is buried at 10 cm depth except for where that depth was not reachable due to large roots or rocks, in which case the cable rests aboveground. Cables also go around tree trunks aboveground and then are buried again on the opposite side. We made similar lines of 10 cm deep cuts in the two *reference* plots to create the same disturbance across all six plots. Past studies have shown that the disturbance associated with installation of heating cables does not affect soil temperature or biological processes such as soil respiration [34]. Due to these past results and the high cost of infrastructure required to implement the experimental treatments and measure response variables, we had only “disturbance controls”, known as *reference* plots, in our study. We waited 15 months between the time of cable installation and turning on of experimental treatments to allow the soil microbes, roots and soils to equilibrate following the disturbance in all six plots.

Both electrical power and data wiring for the heating and sensor systems are distributed in a system of PVC conduit laid on the surface of the ground running from the equipment shed to the four heated plots (Fig 1). The cables are powered by a 240 V electrical supply, which is controlled by a Campbell Scientific datalogger (CR1000, Logan, UT). The datalogger recorded temperature data from temperature sensors (i.e., thermistors; Betatherm type 10K3A1; CSI part #13424) buried in each heated plot and in reference locations ~2 m outside each of the four heated plots, hereafter referred to as “control sensors” (*n =* 2 control sensors per plot). Temperature data from control sensors and reference plots are used to control when the heating cables are turned on or off, similar to a thermostat in a home heating system. The cables apply continuous heating until the target temperature difference (5°C above reference conditions) is achieved. Thermal images of the plots show that soils are warmer immediately adjacent to heating cables.

Electrical power wiring is run between plots to minimize wire lengths and data wiring is run along the outside of the plots to separate data wiring from the power wiring so as to minimize electrical interference with data signals (Fig 1). All wiring splices and junctions are made in weather-proof PVC boxes via terminal strips. At each heated plot there are two power distribution junction boxes for the heating cables and all six plots also have data junction boxes for sensors. To the greatest possible degree, sensor wiring is installed in conduit within the plot to minimize the likelihood of damage.

The experimental warming applied to the four heated plots during the snow-free season is intended to simulate an anticipated 5°C rise in air temperatures by the year 2100 [35]. At the onset of the snow-free season, defined here as the time at which all snow and soil frost had melted and the daily soil temperatures began a natural increase tracking air temperatures [36], we began soil warming in both the *warmed* and *warmed + FTC* plots. This onset of snow-free season warming occurred on April 18, 2014 and April 21, 2015 in each treatment year. Once triggered, the heating cables were used to maintain soil temperatures at 5°C above *reference* soil temperatures throughout the snow-free season and continue until the mean air temperatures are below 0°C for five consecutive days or December 1, whichever comes first, to avoid warming throughout the winter in the event of a low snow year. Warming cables were turned off at the end of the snow-free seasons on November 19, 2014 and December 1, 2015. The annual power consumption to maintain the experimental treatments and sensors in 2015 was 63,521 kWh.

During the first snow event of each winter, snow was packed down gently in the *warmed + FTC* plots with the back of a shovel to create a 3 to 5 cm base layer to minimize disturbance during subsequent shoveling and maintain the winter albedo of the forest floor. We induced each soil FTC by removing snow via shoveling within 48 hours of snowfall to induce a 72-hour soil freeze event (defined as soils at or below– 0.5°C for 72 hours), followed by warming with heating cables to 1°C to induce a 72-hour thaw. During each shoveling event, we did not penetrate the base layer of snow to avoid disturbing the forest floor; all snow that was removed was deposited at least at a meter outside of the *warmed + FTC* plots. The 72-hour thaw did not melt the base layer of snow packed down during the first snow event of each winter. Soil temperatures in all six plots were streamed in real-time at http://hbrsensor.sr.unh.edu/data/soilwarm and monitored remotely to determine when each 72-hour freeze event was complete to initiate the 72-hour thaw. The real-time view of soil temperature data is critical for control of warming cables. After the 72-hour thaw, the heating cables were turned off. We induced four FTC each in the winters of 2013/2014 and 2014/2015 (Table 2).

**Table 2:** Winter environmental variables by experimental treatment averaged across both winters of 2013/2014 and 2014/2015. Minimum soil temperatures at 10 cm depth shown below. Values are means with standard error (*P < 0.05, ** *P* < 0.0001 for comparisons among treatments; degrees freedom = 2). Distinct letters within a row indicate statistically significant differences among treatments.

	*Reference*	*Warmed*	*Warmed + FTC*
Minimum Soil Temperature (°C)**	-0.09^a^ ± 0.04	-0.09^a^ + 0.09	-2.49^b^ ± 0.31
Maximum Snow Depth (cm)**	63.9^a^ ± 5.4	57.6^a^ ± 5.0	24.2^b^ ± 4.1
Snow AUC (cm days)**	4065.6 ^a^ ± 202.1	3766^a^ ± 327.1	714.9^b^ ± 29.6
Maximum Frost Depth (cm)**	12^a^ ± 1.1	12.7^a^ ± 1.0	21.5^b^ ± 1.1
Frost AUC (cm days)*	1108.2 ^a^ ± 71.8	1209.1^a^ ± 66.0	1442.4^b^ ± 69.7
Number of Soil Freeze-Thaw Cycles	0	0	4

### Response variables

We installed thermistors and soil moisture probes (model CS616, Campbell Scientific, Logan, UT, USA) in each of the six plots in fall 2012. The soil moisture probes were inserted vertically to provide an integrative measure of soil moisture in the top 30 cm of soil. Within the two *reference* plots, there are two soil moisture probes, four thermistors at 10 cm depth, two thermistors at 5 cm depth, and one thermistor at 30 cm depth. In each of the two *warmed* and two *warmed + FTC* plots, there were four moisture probes, six thermistors at 10 cm depth, two at 5 cm depth, and one at 30 cm depth. The *warmed* and *warmed + FTC* plots had two more thermistors than the *reference* plots in order to better capture the variability in soil temperature induced by the warming cables. We installed two relative humidity and air temperature sensors (both at height of 6 m above the forest floor; model CSI 215 Campbell Scientific, Logan, UT, USA). There are six photosynthetically active radiation (PAR) sensors (model SQ110, Apogee Instruments, Logan, UT): one in the center of each of the six plots at 1 m height above the forest floor. Temperature, moisture, relative humidity, and PAR data are recorded every 30 seconds, averaged every half hour, and stored in a CR1000 multichannel data logger (Campbell Scientific, Utah, USA). Plots were divided into four equal sized quadrants and the probes were installed evenly across each plot to have an equal distribution of measurements across each quadrant. For example, one moisture probe was installed at the center of each quadrant, making four total per plot. Probes were installed between cables and at least one meter from the nearest tree stem.

Snow depth was measured weekly in four locations in each plot from December through April using a meter stick placed vertically into the snowpack on each sample date in each plot quadrant. Soil frost depth was measured between the surface of the forest floor and the maximum depth of frozen soil weekly using frost tubes constructed of flexible PVC tubing (1.3 cm diameter) filled with methylene blue dye [37], which were inserted into rigid PVC casings 50 cm below the soil surface at four locations within each plot. We installed one frost tube in each plot quadrant and all tubes were placed at least two meters from the plot edge and within one meter of a thermistor. We calculated an integrated metric of depth and duration of snow and soil frost by summing the "area under the curve" (AUC) with either soil frost or snow depth, respectively, along the y-axis and sampling date along the x-axis [38]; R pracma package, V 1.8.8, [39]).

### Statistical analyses

The effects of experimental treatment on snow-free season soil temperature, snow-free season soil moisture, and winter environmental variables were assessed by plot using linear mixed effects models with plot nested within year as the random effect (R nlme package V 3.1–124, [40]). Post-hoc pairwise comparisons among treatments were calculated using Tukey's HSD tests (R multcomp package, [41]). Assumptions of normality and constant variance were assessed by visual inspection of residual plots and data were log transformed when necessary. All statistical analyses were conducted in R version 3.1.2 (R Core Team 2014). Unless otherwise noted, values presented are treatment means with standard error.

## Results and discussion

**Fig 2:**
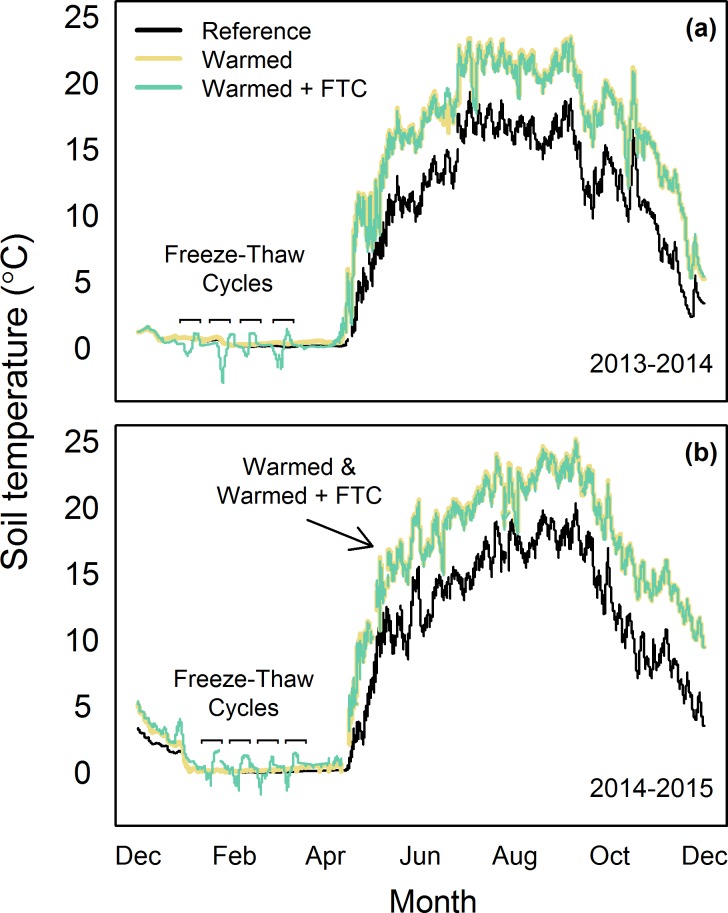
**Soil temperatures from a) December 2013 to December 2014 and b) December 2014 to December 2015.** Soil temperatures averaged by treatment for 10 cm depth thermistors for each day.

**Fig 3:**
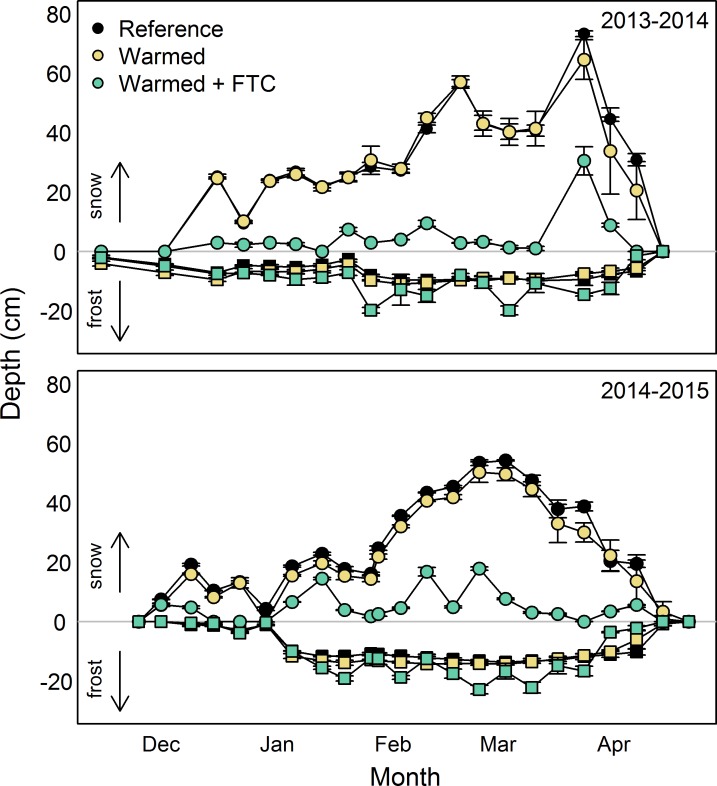
Mean snow (>0 cm, circles) and frost depth (<0 cm, squares) in *reference*, *warmed*, and *warmed + FTC* plots. Error bars are standard error of the mean. Upper figure contains data from the 2013/2014 winter and lower figure contains data from the 2014/2015 winter.

**Fig 4:**
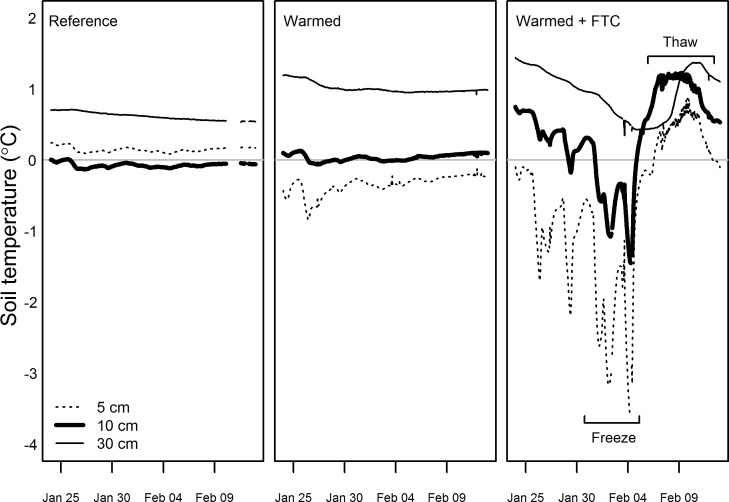
Soil temperatures averaged by treatment for each depth (5, 10, and 30 cm) during one freeze-thaw cycle in winter 2015 in the *reference* (left), *warmed* (center), and *warmed + FTC* treatment (right). We used soil temperatures at 10 cm depth (thickest line) to operationally define one soil FTC. Note that while the temperatures at 5 cm depth are higher than 10 cm depth in the *reference* plots, they are not significantly different from each other.

By employing a combination of buried heating cables and snow-removal, we achieved a 5°C increase in average soil temperatures in the snow-free season and four soil FTCs in each winter, both of which are consistent with projected changes in temperature for northeastern U.S. forests over the next century [35]. Because soil temperatures track air temperatures during the growing season, we feel confident using 5°C as the expected increase in soil temperatures over the next century, as projected for air temperature in a variety of climate model scenarios [35]. The snow-free season warming treatment led to increased mean soil temperatures in the *warmed* and *warmed + FTC* treatments relative to the reference treatment by 4.9 ± 0.8°C in 2014 and 2015 at 10 cm depth (all *P* < 0.0001; Fig 2). Soil temperatures at 30 cm depth in the *warmed* and *warmed + FTC* treatments relative to the reference plots were elevated by 4.9 ± 0.0°C in 2014 and 4.5 ± 0.2°C in 2015 at 30 cm (*p =* 0.02). Soil temperatures declined with depth across all treatments in the snow-free seasons of both 2014 and 2015 (*P* = 0.023 in 2014 and < 0.0001 in 2015). Mean winter soil temperatures in the *warmed + FTC* plots were not different from the other plots (*P* > 0.05), but the *warmed + FTC* plots had lower minimum soil temperatures, snow depth, and snow AUC, as well as deeper maximum soil frost depth, greater soil frost AUC and more soil FTCs than the other four plots (Table 2; Figs 2 and 3). The greater maximum depth of soil freezing in the *reference* and *warmed* plots (i.e., 12 cm) compared to the long term average (i.e., 6 cm) measured in multiple locations throughout Hubbard Brook is likely due to local spatial variation in soil freezing. The effects of induced freeze-thaw cycles on soil temperatures varied by depth and influenced soils to a depth of at least 30 cm, while soil temperatures in *reference* and *warmed* plots remained relatively constant and near freezing (Fig 4). These changes in winter dynamics illustrate that the experimental treatments at CCASE lead to increased variability in winter soil temperatures through induction of soil FTCs, without affecting mean temperatures. Together, the changes in winter conditions in the *warmed + FTC* plots mimic projected changes in soil thermal regimes for the northeastern U.S. [6] more accurately than snow removal alone would. While other experiments have successfully induced realistic increases in winter temperature variability in small statured ecosystems such as grasslands using infrared lamps [20, 21, 26], this study is the first that we are aware of to document a similar treatment in an intact tall statured forest ecosystem.

**Fig 5:**
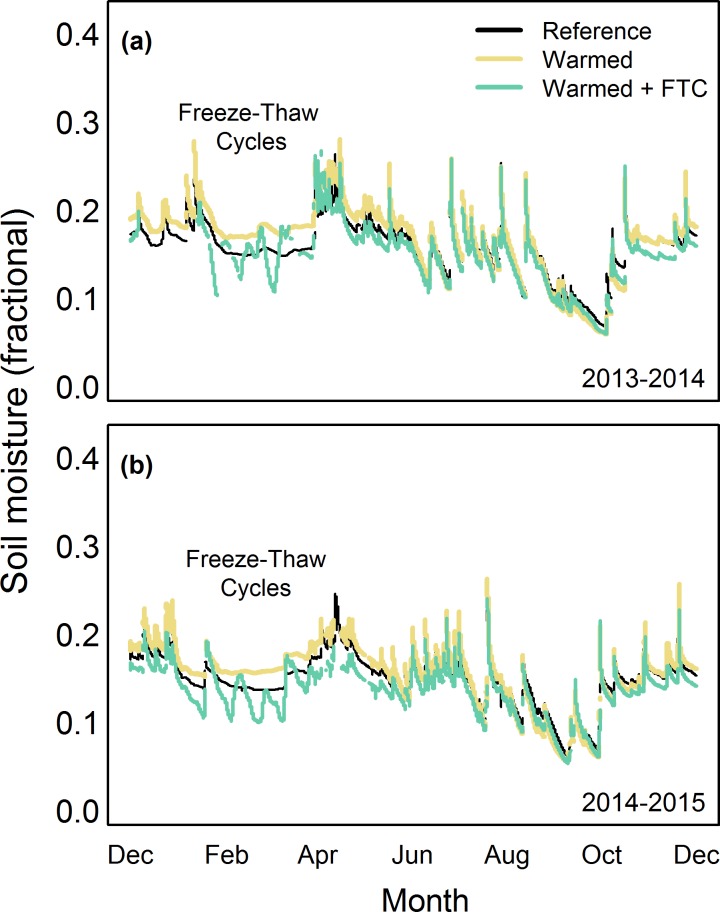
Soil moisture integrated from 0–30 cm depth from a) December 2013 to December 2014 and b) December 2014 to December 2015 averaged by treatment for each day.

Soil moisture between 0–30 cm depth was not significantly different among treatments in the snow-free seasons of 2014 (*P* = 0.71) and 2015 (*P* = 0.64) or the winters of 2013/2014 (*P* = 0.39) and 2014/2015 (*P* = 0.26; Fig 5), which is similar to some experiments [42] and unlike many other experiments that document warming-induced drying of soils [43]. The lack of warming effect on soil moisture suggests that drying artefacts associated with many other warming experiments are not present in this study, at least in the first two years of treatment. We acknowledge that warming effects on soil moisture might be more apparent with more refined depth measurements of soil moisture. Our moisture sensors integrated soil moisture over the top 30 cm of soil and it is possible that measuring soil moisture at multiple depths independently may show effects of warming at some depths and not others, as was observed when monitoring soil temperature at 5, 10, and 30 cm soil depths (see Fig 4).

To our knowledge, no other climate change experiment examines the combined effects of distinct projected changes in soil temperature across seasons (i.e., warmer soils in the snow-free season combined with greater frequency of soil FTCs in winter) or has induced multiple soil FTCs that match the projected rise in frequency over the next century in an intact tall statured forest ecosystem. Previous experiments in seasonally snow-covered ecosystems that have only warmed soils [9] or manipulated snow [10] are unable to capture the combined effects of projected changes in winter and snow-free season climate in seasonally snow covered ecosystems. The unique experimental design of CCASE provides scientists with a new approach to manipulating ecosystem soil thermal regimes that more closely simulate projected changes in temperature across seasons. This experiment provides the basis for long-term monitoring of ecosystem and biological responses to climate change, which is essential for providing insight into ecosystem dynamics over time [14, 44]. Together, these measurements will be used to infer the combined effects of rising air temperatures occurring during both winter and the snow-free season on forest nutrient retention, transpiration, microbial and arthropod abundance and diversity, and carbon sequestration.

We demonstrate proof of concept for studying ecosystem responses to climate change across seasons in tall-statured forest ecosystems, which are often the most difficult terrestrial ecosystems to manipulate. The experimental design of CCASE is easily scalable and transferable to other seasonally snow-covered ecosystems such as boreal peatlands, arctic and/or alpine tundra, and grasslands that are also likely to experience soil warming during the snow-free season and changes in soil freezing dynamics during winter. By integrating projected changes in climate across seasons, future climate manipulation experiments can more accurately characterize ecosystem response to climate change across the 45–57% of northern hemisphere land area influenced by seasonal snow cover and soil freezing dynamics [1].
